# A single-phase seven-level switched capacitor with common ground inverter and improved phase-shift modulation technique

**DOI:** 10.1038/s41598-025-86180-y

**Published:** 2025-02-04

**Authors:** Bisma Saif, Adil Sarwar, Shafiq Ahmad, Shiue-Der Lu, Hwa-Dong Liu

**Affiliations:** 1 Department of Electrical Engineering, ZHCET, Aligarh Muslim University, Aligarh, 202002 India; 2 Industrial Engineering Department, College of Engineering, King Saud University, 11421 Riyadh, Saudi Arabia; 3 Department of Electrical Engineering, National Chin-Yi University of Technology, Taichung, 411 Taiwan; 4 Undergraduate Program of Vehicle and Energy Engineering, National Taiwan Normal University, Taipei, 106 Taiwan

**Keywords:** Multi-level inverters (MLIs), Mean time to failure (MTTF), Mean time before failure (MTBF), Improved phase shift (IPS), Phase shift (PS), Switched capacitor (SC), Total harmonic distortion (THD), Energy science and technology, Engineering

## Abstract

Lately, transformer-less Researchers in the fields of power electronics and renewable energy have taken notice of photovoltaic inverters because of their great efficiency, low cost, and small size. However, higher efficiency typically results in more components, making the inverter costly and bulky. This article proposes a single-phase seven-level transformer-less with common ground topology. The proposed topology utilizes 10 switches, 4 capacitors and 1 diode. This article also suggests an improved Phase Shift (PS) Modulation Technique which reduces overall losses. When implemented with improved Phase Shift (PS), the total highest attainable efficiency of the proposed topology is 98.05% at 15 W. The THD% of voltage harmonics is reduced to 15.29% from 17.20% and for current harmonics is reduced to 5.07% from 10.15%. The reliability of the proposed inverter has also been analyzed. The simulation as well as hardware results have been presented to validate the performance of the proposed inverter.

## Introduction

A Multilevel Inverter (MLI) is commonly employed in applications that require high voltage and high current.

The desired stepped AC voltage waveform is derived from many levels of DC voltage. Using a multi-level inverter offers several advantages over a traditional two-level inverter, including reduced switching losses and frequency. As the number of levels increases, the total harmonic distortion (THD) decreases. However, this level increase is accompanied by a substantial increase in the number of components. As the number of components in an inverter increases, the inverter gets larger, more expensive, and less efficient, so negating its advantages^1^.

Among all renewable energy sources, solar photovoltaics (PV) is one of the purest, most dependable, emission-free, and most readily accessible. PV generation has grown significantly in both business and residential buildings in recent years. Global solar power generation reached 1270.5 GW by the end of 2022, predicted Solar Power Europe’s global market projection for 2018–2022. It was anticipated to reach 209 GW in China and 78.4 GW in India^2^. Power electronic converters are essential for integrating renewable energy sources with the utility system. The two steps of a typical grid-connected photovoltaic system are dc–dc and dc–ac conversion. A dc–ac conversion stage is required for grid-tied PV systems in order to input AC electricity into the utility grid. The two main categories for these grid-tied inverters are galvanic-isolated systems and non-isolated systems^2,3^.

To overcome the above issue, higher efficiency, and low cost, several transformer-less inverters (TIs) with inherent voltage boosting ability are proposed in the literature^3,4^. However, the absence of galvanic isolation and high-frequency switching excites the ground leakage current as the PV panels are grounded to meet electrical codes and safety standards. These topologies lower the leakage current by either connecting the grid neutral to the midpoint of the dc-link bus, isolating the PV array from the utility grid to maintain a constant common mode (CM) voltage, or connecting the grid neutral to the negative terminal of the PV array^4,5^.

Significant efforts are needed to control the leakage current by structural alterations in order to maintain a consistent common mode voltage. On the other hand, researchers have been able to build a number of inverter topologies utilizing this approach because a common ground connection can prevent ground leakage current^6^.

Since all components share the same ground, there is less potential for ground loops, which can introduce noise, reduced noise, and electromagnetic interference (EMI). Components and subsystems can be easily interfaced with each other when they share a common ground. This is particularly useful in complex systems where multiple modules are needed to communicate or share signals. These are just a few of the advantages that make this topology so widely used in a wide range of applications.

By establishing a common ground connection between the PV negative line and grid neutral, the common ground type (CGT) inverter eliminates leakage current and avoids the PV parasitic capacitance. While typical CGT-based inverters do not use leakage current, they are more expensive and need more volume since they use at least one capacitor rated for maximum output voltage. Larger voltage-rated capacitors are needed for the majority of CGT inverters that are currently on the market, which increases ripple losses and energy storage. Even if some of the less complex capacitor-stored energy-based CGT topologies handle this problem, more compact and simplified structures can still be created^7,8^.

The evolution of power electronics topology has been transformative, driven by advancements in efficiency, power density, and component optimization to meet the demands of modern high-power applications. The field initially relied on conventional DC–DC and DC–AC converter topologies, such as buck, boost, and basic inverter designs, which allowed basic energy conversion with limited efficiency. As technology progressed, the need for better power quality and reduced switching losses spurred the development of multilevel inverters (MLIs), which include topologies like Neutral Point Clamped (NPC), Flying Capacitor, and Cascaded H-Bridge inverters. These topologies improved output waveform quality, reduced harmonic distortion, and mitigated voltage stress on power switches, resulting in more efficient and durable designs suitable for high-voltage, grid-connected applications^9,10^.

Recent years have witnessed substantial research into optimizing power converters with reduced component count and simplified structures. Innovations in switch reduction strategies reflect significant progress toward achieving high efficiency with fewer components. Such designs not only improve system efficiency but also reduce costs and complexity, making them ideal for medium- and high-voltage applications^11^.

The hybridization of inverter topologies has also introduced adaptive designs that combine elements from different MLIs, such as the Hybrid Cascaded H-Bridge and Modular Multilevel Converters (MMCs), which are particularly advantageous for renewable energy integration and DC transmission. Hybrid inverters offer scalability, reduced harmonic content, and enhanced control flexibility, achieving efficiencies up to 97.8% at full load while operating with lower voltage stress on components^12,13^. MMCs have furthered this progression by offering modularity, fault tolerance, and scalability, which are essential for the evolving landscape of grid-tied and high-voltage direct current (HVDC) systems, where power quality and reliability are paramount^12^.

More recently, the adoption of wide-bandgap (WBG) materials like Gallium Nitride (GaN) and Silicon Carbide (SiC) has been a game-changer in topology development. These materials enable higher switching frequencies and lower conduction losses compared to traditional silicon-based devices. For example, SiC-based NPC topologies have achieved up to a 3% improvement in efficiency at switching frequencies exceeding 50 kHz, a milestone in high-frequency power applications that require minimal loss and high durability. Looking forward, AI-driven adaptive switching mechanisms are emerging as a cutting-edge addition to power electronics, enabling real-time adjustments to optimize performance across variable loads. This evolution from basic converters to intelligent, high-efficiency topologies reflects the ongoing pursuit of power density, cost reduction, and control precision to address the complex requirements of modern power systems and renewable energy applications^13^.

Modulation techniques for multi-stage inverters are diverse. A multilayer inverter uses the modulation approach to obtain the regulated output voltage and so synthesizes a sinusoidal waveform.

Phase Shift Modulation (PS) is a modulation technique in which the digital signal data modifies the carrier signal’s phase. Because of PS’s dependability and effectiveness, digital communication systems employ it extensively. The main benefit of PS modulation is that it can withstand noise and interference, which makes it appropriate for situations where there is a lot of signal distortion. This robustness results from the information being transmitted through phase shifts, which are less vulnerable to noise-induced amplitude alterations.

When Phase Shift Modulation (PS) is implemented in DC–DC inverters, the system can experience high losses primarily due to switching and conduction inefficiencies. In PSM, the output voltage is regulated by adjusting the phase shift between the control signals driving the inverter’s switches. This modulation technique typically involves high-frequency switching to achieve precise control over the power flow, which inherently increases switching losses. Each transition from on to off state (and vice versa) of the power transistors generates switching losses, proportional to the switching frequency and the voltage and current during the transition. Higher switching frequencies exacerbate these losses^14,15^. In an attempt to eliminate these drawbacks, many methods of improving phase shift modulation have been proposed.

Recently, many multi-level topologies have been proposed. Jakhar et al.^16^ proposed a 7-level single phase topology which utilizes around 12 switches and 6 capacitors, with efficiency up to 95%. The main drawback in the proposed topology here is that the proposed topology uses a higher component count, providing less efficiency. This reduced component count not only lowers costs but also simplifies installation, also reducing thermal stress for medium voltage systems. Meraj et al.^17^ proposed a dual circuit. For seven level circuit proposed it utilizes 12 switches and 2 capacitors giving 94% efficiency. The design also utilizes H-bridge for extension of levels. The proposed circuit utilizing around 10 switches, and 4 capacitors provides 98.05% efficiency at lower loads, making it reliable for medium voltage circuits. Meraj et al.^18^ proposed a 9-level inverter topology utilizing 12 power switches (10 switches, and two of which are bidirectional) 2 capacitors and also 2 DC sources, providing 94.5% efficiency and lower for varying loads. One of the main drawbacks is the use of two DC sources, which requires high input voltage overall increasing voltage stress. Although it gives a higher number of levels, its losses are still comparatively high. Neti et al.^19^ proposed a 5-level CGT utilizing 7 power switches (6 switches, out of which one is bidirectional). The topologies proposed above are mainly, with high component count, high voltage stress and less efficiency when compared to the proposed topology.

The researchers have developed many multilevel inverters that give several voltage levels and high gain. As many aim to achieve high gain, they cannot handle the increasing voltage stress on the components and thus reduce overall efficiency. The topologies proposed in^20^ are 19-level ANPC-SCMLI inverters, which utilize four switched capacitors and two DC link sources. The highest efficiency achieved is 94.42% at 500W. The topology provides a very high gain, which results in high voltage stress and thus reduces efficiency. The topology proposed in^21^ is a 5-level CGT, utilizing 8 switches and two capacitors with an efficiency of 97.2%, giving a dual boost. The topology proposed in^1^ is a 17-level inverter that utilizes 5 half-bridge switches, 4 capacitors, and 5 diodes, providing an efficiency of 95.4%. The topology proposed in^13^ is a 5-level CGT- inverter that can be extended to further higher levels; its highest attainable efficiency is 96.20%. It utilizes 8 switches and 2 capacitors. The topologies proposed have a high voltage gain and require less input voltage, which is of great significance. The high gain and low input create a higher voltage stress on switches used, which in turn impacts the performance and efficiency of the inverter. Another 13-level topology has also been recently proposed in^13^, which utilizes 15 switches and three capacitors and has an efficiency of about 97%. The topologies proposed here all are of higher or lower level, providing higher gains.

The major drawback of all the topologies mentioned above is voltage stress or component count.

The design of the proposed inverter and the implementation of the proposed modulation scheme help in reducing losses and enhancing efficiency, providing a triple boost. This paper proposes a seven-level CGT inverter with a reduced component count, less bulky, cost-effective with enhanced efficiency and an improved phase shift modulation technique which gives a highest attainable total efficiency of 98.05%, reducing both switching and conduction losses-.

The features of this paper are:

- Proposed topology with reduced number of components and reduced THD.
- Proposed an improved phase shift modulation resulting in lower losses and enhanced efficiency.
- Overall enhanced efficiency when Improved phase shift is implemented on the proposed topology.

## Proposed Seven-level inverter and improved phase shift (IPS)

This section discusses the proposed topology, its operating principle and improved phase shift modulation technique.

### Proposed technology

**Fig. 1:**
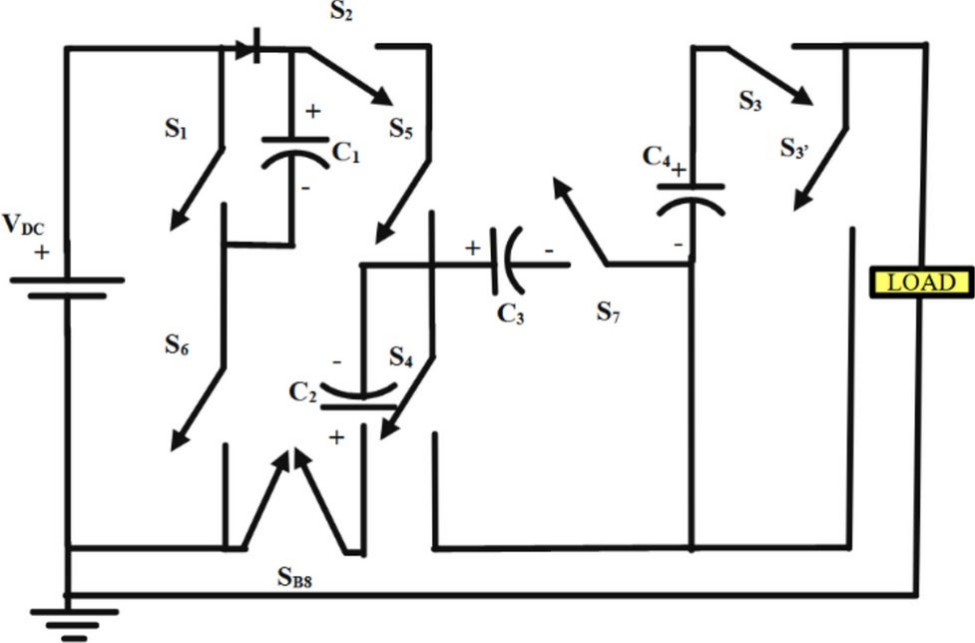
Proposed circuit.

The proposed seven-level inverter has 10 power switches, of which two are connected antiparallel making a bidirectional switch, S_B8_, 4 capacitors, two of which are charged to 1V_DC_ and the other two to 2V_DC_, and 1 diode. Figure 1 shows the proposed circuit.

The circuit consists of 4 switch legs, leg 1 consisting of S_1_ and S_6_, leg 2 consisting of S_4_ and S_5_, leg 3 consisting of S_3_ and S_3’_and leg 4 consisting of S_7_ and S_B8_.

### Operation principle

**Fig. 2:**
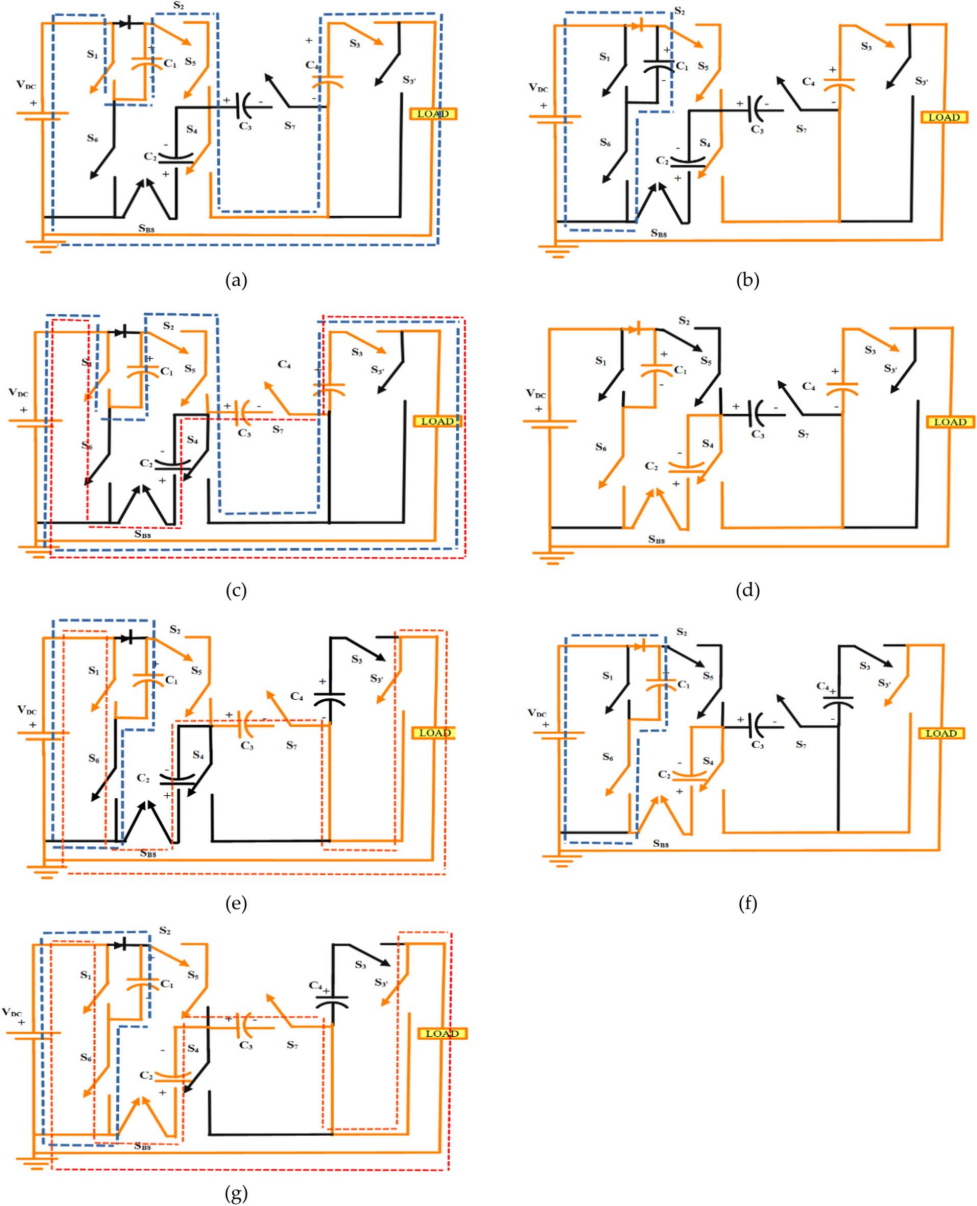
(**a**) + 3V_DC_, (**b**) + 2V_DC_, (**c**) + 1V_DC_, (**d**) 0V_DC_, (**e**) − 1V_DC_, (**f**) − 2V_DC_, (**g**) − 3V_DC_.

Figure 2 shows the various operating modes of the proposed circuit. C_1_ and C_2_ are charged to 1V_DC_ whereas C_3_ and C_4_ are charged to 2V_DC_. S_B8_ allows current to flow in either direction, helping in generating ± 3V_DC_ levels.

Table 1 shows the switching states of all the switches for generating various levels.

**Table 1:** Switching states.

Voltage levels	S_1_	S_2_	S_3_	S_3_^’^	S_4_	S_5_	S_6_	S_7_	S_B8_
+ 3V_DC_	1	1	1	0	1	1	0	0	0
+ 2V_DC_	0	1	0	1	1	1	0	0	0
+ 1V_DC_	1	1	1	0	0	1	0	1	0
0V_DC_	0	0	0	1	1	0	1	1	1
− 1V_DC_	1	1	0	1	0	1	0	1	0
− 2V_DC_	0	0	0	1	1	0	1	0	1
− 3V_DC_	1	1	0	1	0	1	1	1	1

As per Table 1, + 3V_DC_ is developed at the output by turning on the switches S_1_, S_2_, S_3_, S_4_ and S_5._ In this state, C_1_ and C_4_ are charged to + 1V_DC_. + 2V_DC_ is developed at output by switching on the switches S_2_, S_3’_, S_4_ and S_5_, here C_1_ is charged to + 1V_DC._ + 1V_DC_ is developed by switching on the switches S_1_, S_2_, S_3_, S_5_ and S_7_. In this state C_1_ and C_4_ are charged to + 1V_DC_ and C_2_ and C_3_ are charged to + 2V_DC_, here the current direction is opposite to the charging of capacitors, thus charging the switches to − 2V_DC_.

0 V_DC_ is developed by turning on switches S_6_, S_7_, S_B8_, S_4_ and S_3’_. − 1V_DC_ is developed at the output by turning on S_1_. S_2_, S_3’_, S_5_ and S_7_. In this state, C_1_ is charged to + 1V_DC_ and C_2_ and C_3_ are charged to + 2V_DC._ The current direction is opposite to charging, so the switches are charged to − 2V_DC_. − 2V_DC_ is developed at the output by turning on S_3’_, S_4_, S_8_ and S_B8_, here C_1_ is charged to + 1V_DC_. − 3V_DC_ is developed at the output by turning on the switches S_1_, S_2_, S_3’_ S_5_, S_6_, S_7_ and S_B8,_ in this state, C_1_ is charged to + 1V_DC_ and C_2_ and C_3_ are charged to + 2V_DC._ The current direction is opposite to charging, so the switches are charged to − 2V_DC._ Orange coloured lines show the current path, the blue dashed lines and red dashed lines show the charging paths of capacitors C_1_ and C_4_ and capacitors C_2_ and C_3_.

### Proposed topology

Switched capacitor multilevel inverters (SCMLI) have several limitations when applied to medium voltage systems. One of the primary issues is the increased switching losses due to the frequent charging and discharging cycles of capacitors. In medium voltage applications, where the inverter must respond quickly to changes in load or operational conditions, these losses can significantly impact overall efficiency and heat generation. Additionally, the challenge of voltage balancing across multiple capacitors becomes more pronounced as the number of capacitors increases, leading to uneven charge distribution. This imbalance can result in capacitor failure or degradation of performance, compromising the reliability of the inverter^22^.

Moreover, traditional SCMLI architecture often requires a large number of capacitors to achieve the desired output voltage levels, which adds complexity to the design and increases the overall size and cost of the inverter. This is particularly problematic in medium voltage applications, where space and weight constraints are critical. Consequently, the limitations inherent in SCMLI designs can hinder their practical implementation in medium voltage systems.

The proposed inverter offers a promising solution to these challenges. By employing a configuration with four capacitors and two bidirectional switches, the design reduces the overall component count compared to traditional MLI topologies. This simplification enhances reliability and minimizes potential points of failure. Additionally, the innovative charging strategy of charging two capacitors to 1 V_DC_ and two to 2 V_DC_ facilitates better voltage control and distribution. Arrangement allows for smoother voltage transitions, thereby enhancing efficiency, as demonstrated by the inverter’s performance of 98.05% at 150W and approximately 97.35% at 1 kW.

Furthermore, this configuration enables the generation of multiple output voltage levels with fewer components, making it particularly well-suited for medium voltage applications. The efficient management of voltage levels not only ensures stable operation under varying load conditions but also reduces the risk of thermal issues commonly associated with high switching frequencies. The ability to maintain high efficiency and effective voltage regulation makes the proposed inverter a reliable option for medium voltage systems.

When compared to other branches of multilevel inverters, such as diode-clamped or cascaded H-bridge inverters, the proposed topology offers advantages in medium voltage scenarios. Diode-clamped inverters often require multiple diodes and capacitors, leading to increased component count and complexity, whereas the proposed topology requires only one diode. Cascaded H-bridge inverters, while modular, still necessitate significant space for multiple H-bridges and associated components, which can be a limitation in compact installations.

### Improved phase shift modulation technique

**Fig. 3:**
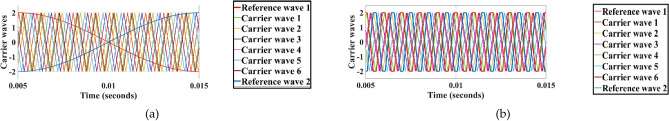
(**a**) Phase shift PWM, (**b**) Improved phase shift PWM.

The Improved Phase Shift modulation technique utilizes Phase Shift PWM, shifting the carrier waves by phase. The reference waves used here to improvise the modulation technique are trapezoidal, resulting in lesser harmonics and, therefore, reduced THD (%) and improved efficiency. Figure 3 shows the phase shift and Improved phase shift modulation techniques.

#### Average switching loss analysis for IPS

1 $$Esw,on = \int_{ton} {i\left( t \right) \cdot v\left( t \right) \cdot dt}$$

2 $$Esw,off = \int_{toff} {i\left( t \right) \cdot v\left( t \right) \cdot dt}$$

The equations given describe the following: i(t) represents the current flowing through the switch; v(t) represents the voltage across the switch; and E_sw,on_ and E_sw,off_ represent the energy losses that occur to the switch during its turn-on and turn-off transition periods, respectively.

Turn-on switching and turn-off switching losses are given by^8^:

3 $$E_{sw,on} = E_{sw,on} *\left( {V_{CC,on} *I_{CC,on} } \right)/\left( {V_{CC,on}^{*} *I_{CC,on}^{*} } \right)$$

4 $$E_{sw,off} = E_{sw,0ff} *\left( {V_{CC,off} *I_{CC,off} } \right)/\left( {V_{CC,on}^{*} *I_{CC,off}^{*} } \right)$$

The values of Esw, on* and Esw, off* in (3) and (4) represent the experimentally recorded energy losses during the transistor’s turn-on and turn-off processes. V_CC_ and I_CC_ represent the maximum voltages and currents required to activate and deactivate the intended state of the switch. V_CC*_ and I_CC_* are the specified voltages and currents for the energy losses that occur when transistor switches are turned on and off. These values can be found in the datasheets of the transistor switches.

By employing the methodology suggested in the reference^8^, we compute the mean switching turn-on and turn-off losses for each switch.

The average switching turn-on power loss is given by^8^:

5 $$P_{sw, on} = \frac{1}{T}\left( {E_{ON,1} + E_{0N,2 } + \cdots + E_{ON,n} } \right)$$

6 $$= \frac{1}{Tp}\left( {E_{ON} \left( t \right)} \right)$$

where,

T = period time considered, T_p_ = period for a single pulse (here for ON state), n = number of switching operations performed during time T.

Similarly, the average switching turn-off power loss is given by:

7 $$P_{sw, off} = \frac{1}{T}(E_{OFF,1} + E_{0FF,2 } + \cdots + E_{OFF,n} )$$

8 $$= \frac{1}{Tp}\left( {E_{OFF} \left( t \right)} \right)$$

where, T = period time considered, T_p_ = period for a single pulse (here for OFF state), n = the number of switching operations performed during time T.

9 $$P_{SW} = P_{sw,on} + P_{sw,off}$$

where, P_sw_ = total average switching loss. T_p_ is calculated utilizing the method mentioned in^8^, similarly, E_on_(t) and E_off_(t) are also calculated using the parameters i_(on)_(t) and i_(off)_(t).

Table 2 presents the different factors used to calculate the average switching losses of each switch. The voltage at the common collector (V_CC_) remains constant for both the turn-on and turn-off durations, as the pulse for each switch has a rectangular shape. The terms "I_CC,on_" and "I_CC,off_" refer to the currents required to turn on and turn off a single pulse. These currents are calculated for a maximum of n switching operations and the power consumption, denoted as P_sw_, is calculated with reference to sources^8^ and^14^. After simplifying the aforementioned process, we can utilize MATLAB Simulink or PLECS to determine the duration of a single pulse for each switch. By referring to the energy loss data provided in the data sheets, we can calculate the average switching losses. However, it is important to note that these calculated values may slightly deviate from the values obtained through power loss analysis using PLECS, due to the inclusion of additional factors such as various losses and thermal considerations. The aforementioned method is an approximation for determining switching losses.

**Table 2:** Parameters provided by the datasheets of the switches used.

Switches parameters used	S_1_	S_2_	S_3_	S_4_	S_5_	S_6_	S_7_	S_B8_
Type of switch	IKW40N 65ES5	IKW40N 65ES5	IKW40N 65ES5	IKW40N 65ES5	IKW40N 65ES5	IKW40N 65ES5	IKW40N 65ES5	IKW40N 65ES5
Rated voltage (V)	400	400	400	400	400	400	400	400
Rated current (A)	4	4	4	4	4	4	4	4
Turn on energy losses (mJ)	0.148	0.148	0.148	0.148	0.148	0.148	0.148	0.148
Turn off energy losses (mJ)	0.069	0.069	0.069	0.069	0.069	0.069	0.069	0.069

## Comparison of proposed topology and improved phase shift (IPS)

### Comparison of proposed topology

In this section, we compare different 7-level CGT inverters to highlight and understand the advantages of the proposed topology. Table 3 discusses switch count, diode count, capacitor count, overall component count and voltage gain of different topologies.

**Table 3:** Comparison of different MLIs with CGT proposed inverter.

Topology parameters	^15^ 2019	^23^ 2020	^24^ 2018	^25^ 2023	^26^ 2023	^27^ 2021	^28^ 2020	^29^ 2024	[PT]
Number of switches	13	10	16	12	11	12	8	6	10
Number of diodes	3	4	–	2	3	0	2	1	1
Number of capacitors	3	2	2	3	3	0	2	3	4
Total component count	19	16	18	17	17	12	12	10	15
Voltage gain	3	3	3	3	3	5	4	4	3
Number of DC sources	1	1	1	1	1	5	1	1	1
Number of levels	7	7	7	7	7	11	11	9	7
Highest efficiency (%)	–	–	–	98	97.85	97.31	94	95.54	98.05

The number of switches used in a topology plays a major role in its efficiency. A lower switch count and higher-level output always result in enhanced efficiency, whereas a higher switch count results in reduced efficiency for it makes the topology bulky and cost ineffective.

Inverters comprising a reduced number of components are expected to exhibit greater efficiency and cost-effectiveness. The second weakest component in power electronic converters is the capacitor. A large amount of the inverter’s bulk, volume, and cost can be attributed to the capacitor. Reducing the number of capacitors and their rating is therefore crucial.

Meraj et al.^15^ utilizes 13 switches, 3 capacitors and 3 diodes for a seven-level output, comparatively higher switch and diode count. The topology discussed in^23^ utilizes 10 switches, 4 diodes and 2 capacitors, while it utilizes the same number of switches, but it utilizes a comparatively higher number of diodes. The topology discussed in^24^ utilizes 12 switches, 4 diodes and 4 capacitors, all components are higher in count making the topology comparatively bulky. The topology discussed in^25^ utilizes 12 switches, 2 diodes and 3 capacitors and discussed in^26^ utilizes 11 switches, 3 diodes and 3 capacitors.

Hosseinpour et al.^28^ utilize 8 switches, 2 diodes and 2 capacitors with total component count of 12 giving 11 levels, but giving an efficiency of only 94%. It gives lesser efficiency due to higher voltage stress on capacitors. Meraj et al.^27^ utilizes 12 power switches but its main drawback is the usage of 5 DC sources, which requires a very high input voltage, reducing efficiency and not making it ideal for medium voltage circuits. The total component count of inverter proposed in^29^ is 10, although less than the proposed, but its efficiency is comparatively less due to voltage stress and design.

The proposed topology [PT] utilizes 10 switches, 1 diode and 4 capacitors, resulting in overall reduced topology and thus enhanced efficiency. A lower capacitor count with a higher rating of the capacitor decreases the efficiency. The proposed topology [PT] consists of 4 capacitors with two capacitors being charged to + 1V_DC_ and two capacitors being charged to + 2V_DC_, the rating of the capacitors is not high, not affecting the efficiency much. The topology also provides a triple boost with higher efficiency.

One of the main reasons for the enhanced efficiency of the proposed inverter is not only due to reduced component count as being compared to few its slightly high, but also reduced voltage stress, two of the capacitors are directly charged from the source to 1V_DC_ without creating much stress and also due to less input voltage required (only 1 DC source), thus increasing efficiency on low loads. Hence the proposed topology achieves qualitative superiority.

### Comparison of IPS PWM and PS PWM

**Fig. 4:**
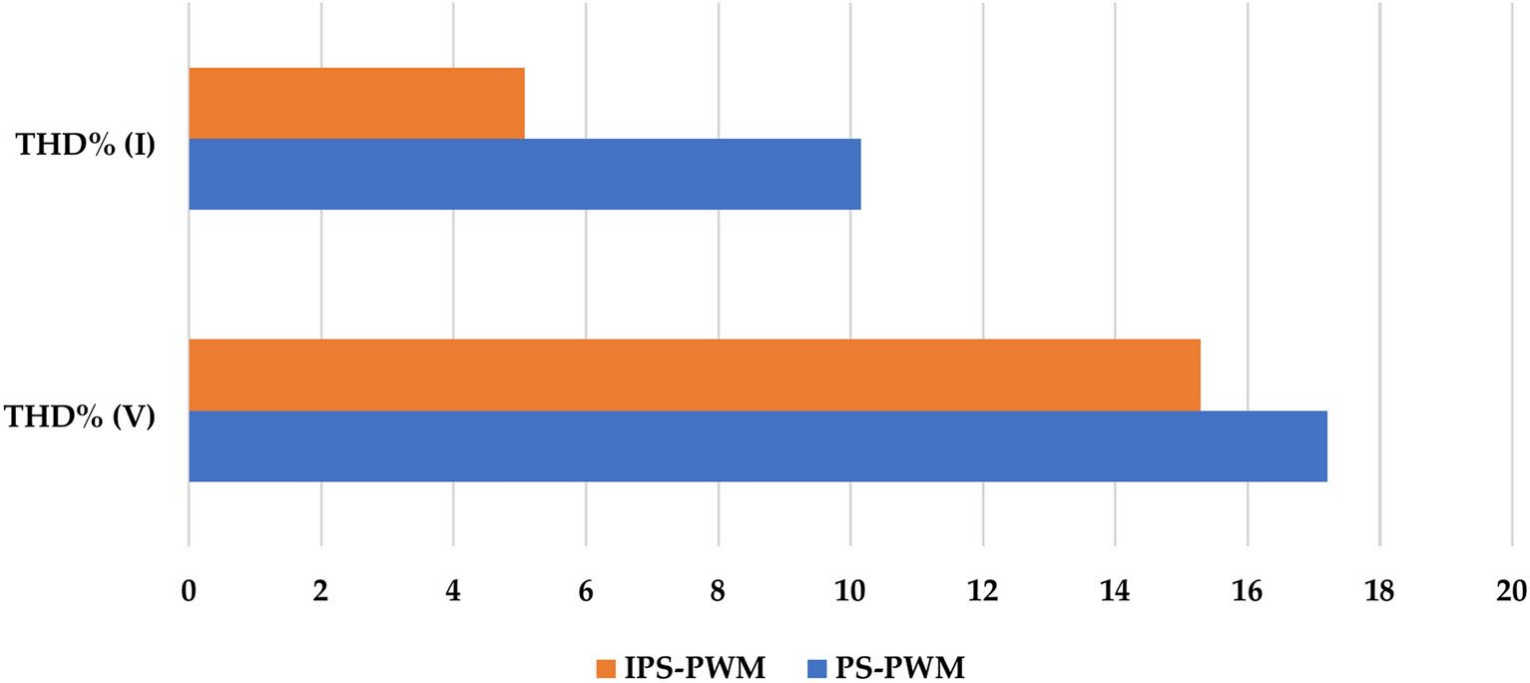
Comparison of voltage and current THD (%).

**Fig. 5:**
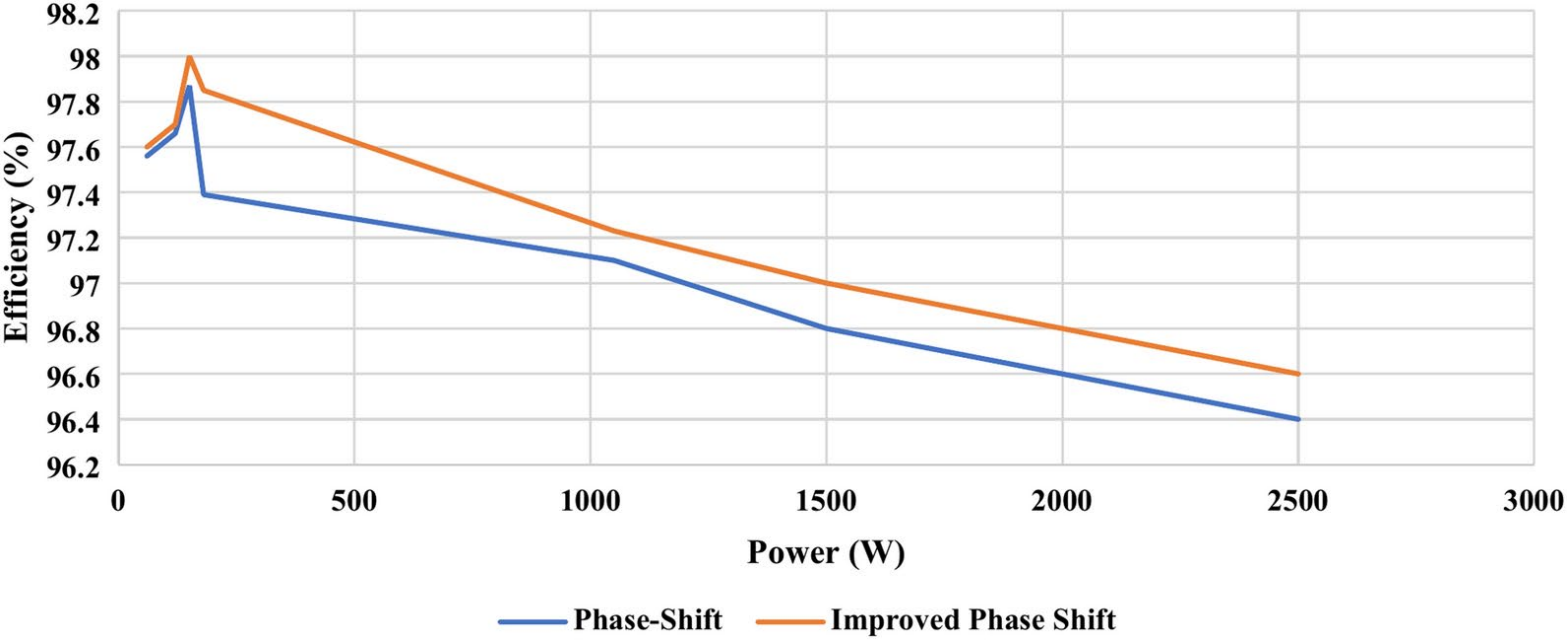
Comparison of efficiency at different loads.

This section compares the PS PWM and IPS PWM in terms of THD(%) and efficiency when implemented on the proposed topology. Figure 4 compares the THD(%) of the two modulation techniques at 500W and 10 kHz switching frequency (Fig. 5).

**Fig. 6:**
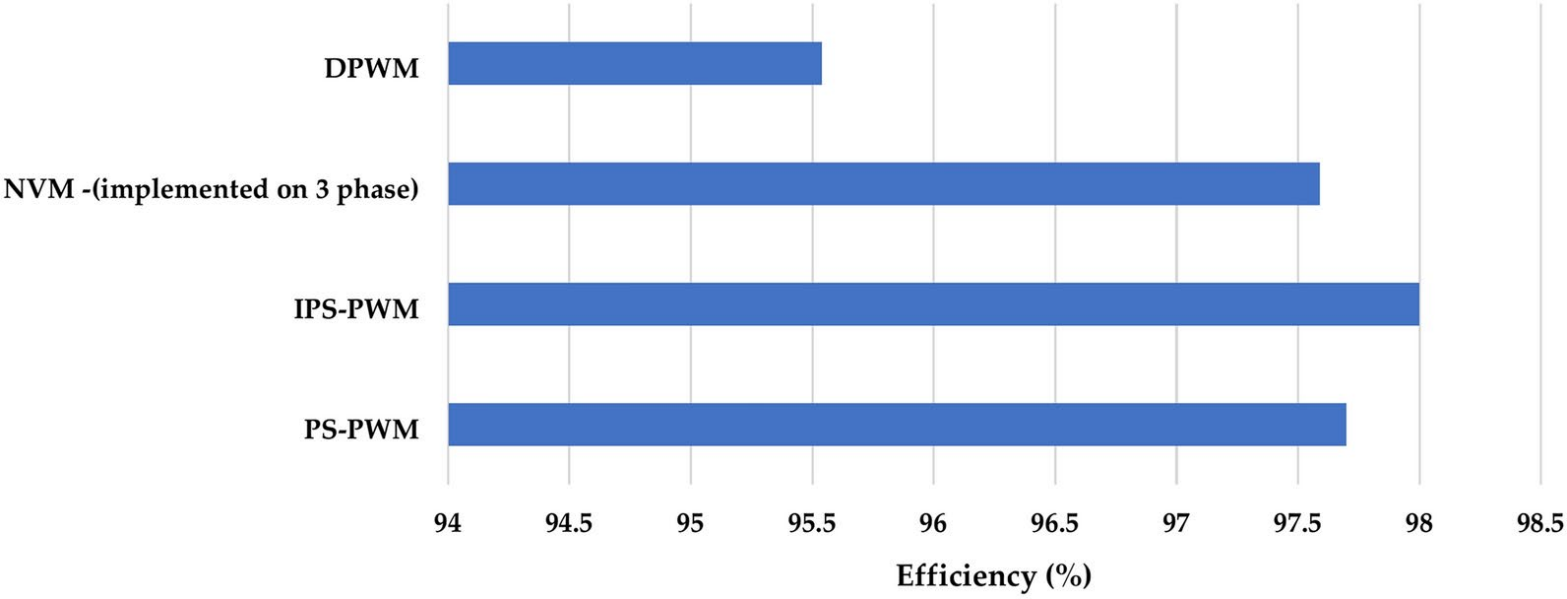
Efficiency of different PWMs.

**Fig. 7:**
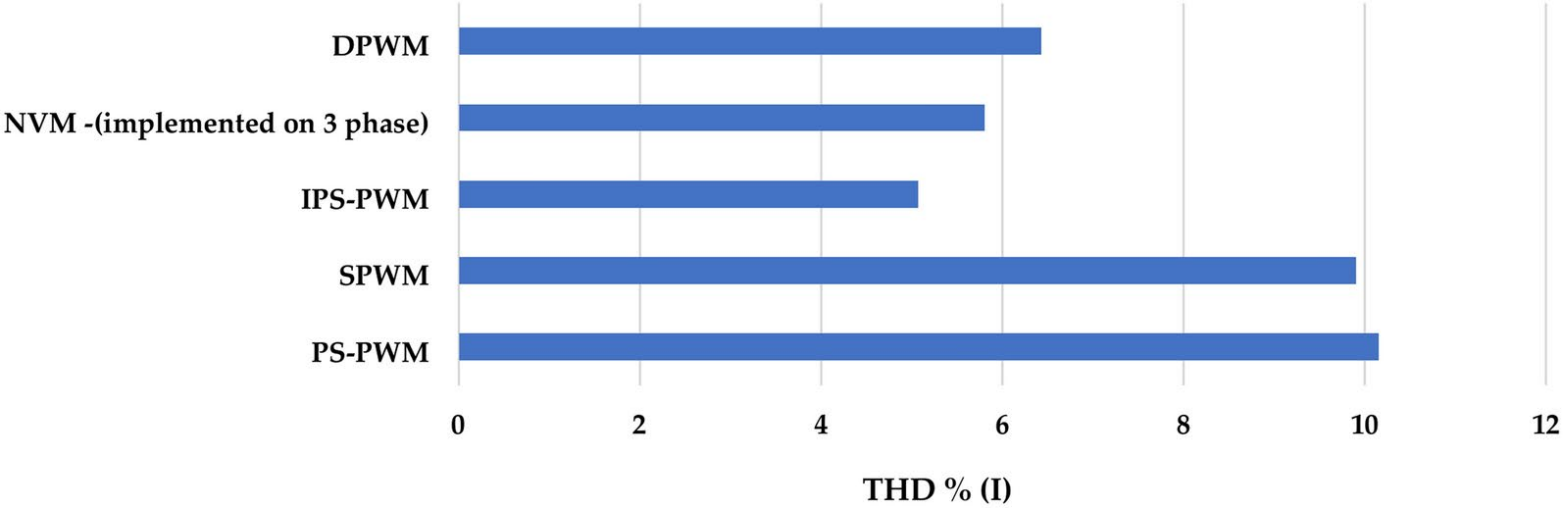
Comparison of current harmonics generated by different PWMs.

Figures 6 and 7 shows the efficiency and THD (current harmonics) comparison of Phase Shift PWM, Improved Phase Shift PWM, Simplified Sinusoidal PWM^30^, hysteresis band-based discontinuous PWM^9^ and Nearest vector modulation PWM^31^. NVM in^31^ has been implemented on a 3-phase inverter, when modulation schemes implemented on 3 phase are most likely to generate lower harmonics and around 5–10% higher when the same is implemented on single phase. From the figure, it can be seen that the IPS PWM generated the lowest THD %.

It is also important to note that the proposed modulation scheme serves the purpose of improving the original Phase Shift PWM, as many advantages are unique to PS PWM only. There are many recent publications of algorithms like^32^ and^33^ when implemented for Selective harmonic Elimination, produce lower THD, as harmonics can be selected, which are desired to be eliminated by the user, but the major purpose served by algorithms for SHE is to eliminate individual harmonics, while IPS PWM serves several advantages which include Modular Multi-Level Topology Support. That is it allows each module (or submodule) within the converter to operate independently in phase, which effectively reduces the switching stress on each individual switch. Moreover, Due to the phase-shifted nature of IPS-PWM, harmonics are naturally attenuated at the output without the need for additional filtering. By phase-shifting the carrier signals for each module, high-frequency harmonics are distributed across a wide spectrum, which results in significant harmonic cancellation at specific frequencies. The proposed modulation scheme not only improvises this quality by reducing THD significantly (compared to PS PWM), it reduces losses and enhances efficiency significantly.

## Power loss analysis

The power losses of an inverter can be imputed for different reasons, including conduction and switching losses induced by other components of the inverter, such as diodes and capacitors. Evaluating power loss is crucial to ensure reliable, economical, and effective operation. The efficiency of the system improves when losses are minimized.

**Fig. 8:**
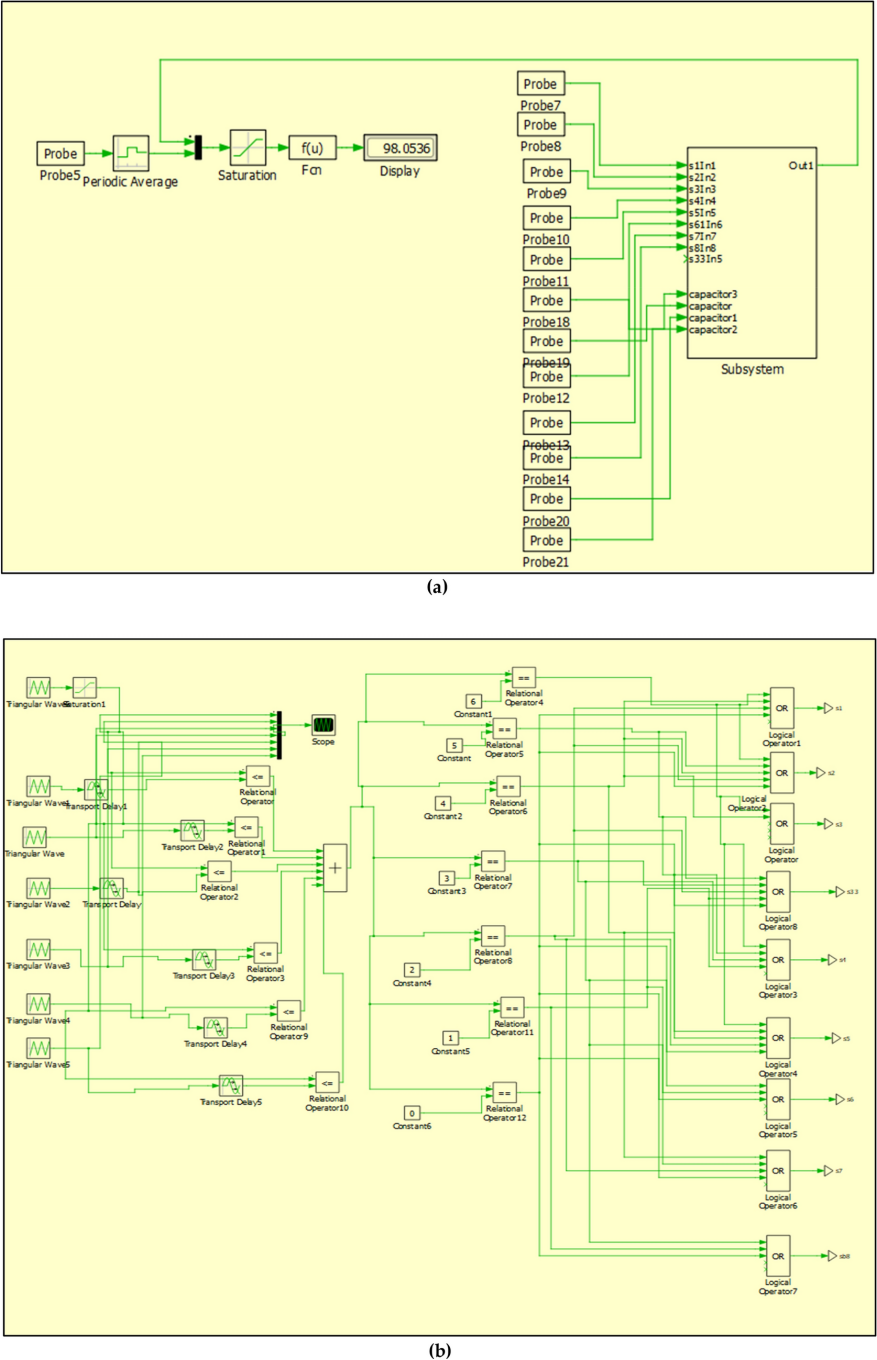

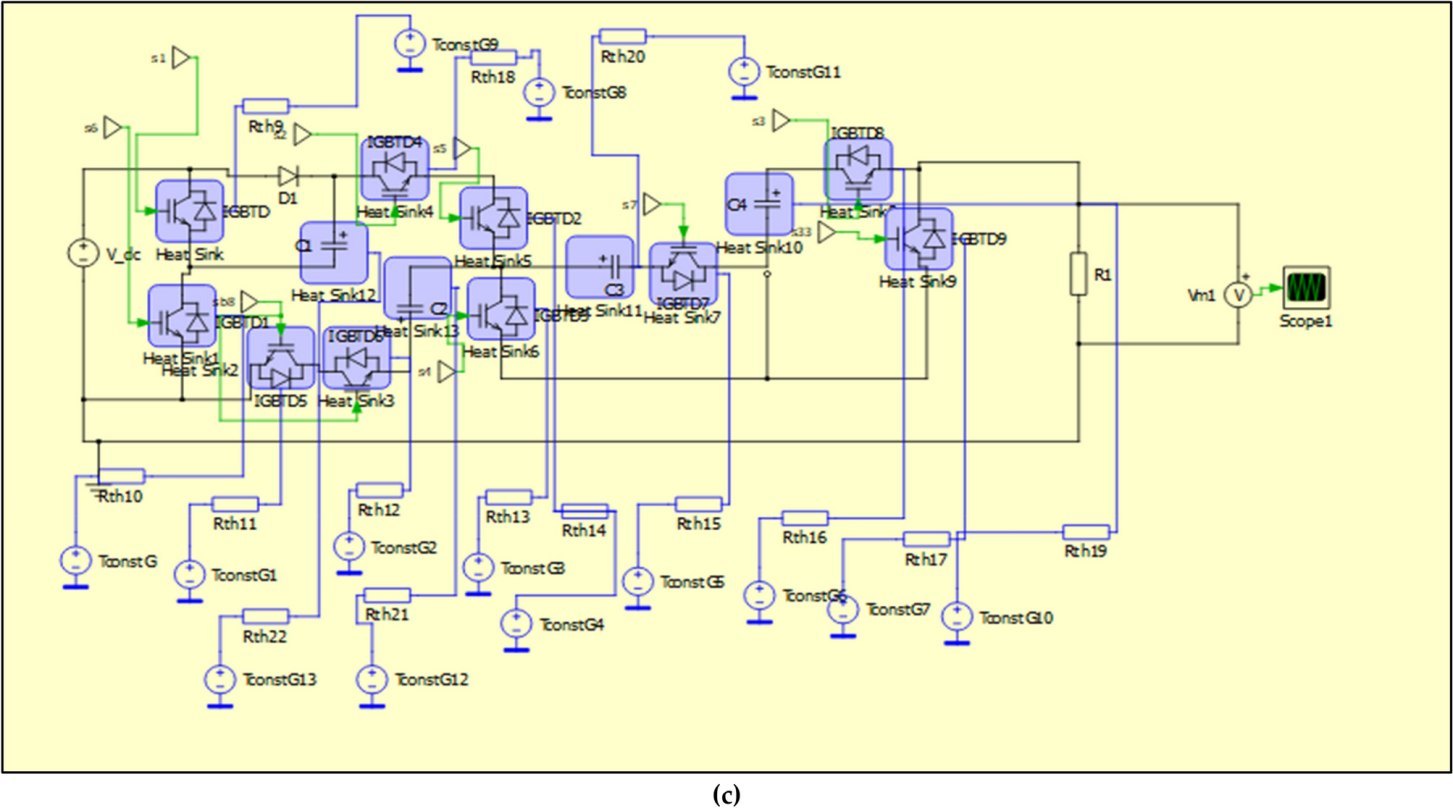
Implememtation of the proposed circuit and modulation scheme to calculate losses and efficiency (**a**) circuit to calculate losses and efficiency (**b**) logic circuit implementing the modulation scheme and switching (**c**) proposed circuit and heat sinks for calculating losses.

In this section, the power loss of the proposed topology when implementing PS PWM and IPS PWM are analysed. This analysis has been performed on the software PLECS. Table 4 provides the required details of the components used when performing the analysis (Fig. 8).

**Table 4:** Specifications of the components used.

Components	Type/specifications
Switches	IKW40N65ES5
Diodes	IKA10N65ET6
Switching frequency	10 kHz
Voltage frequency	50 Hz

**Fig. 9:**
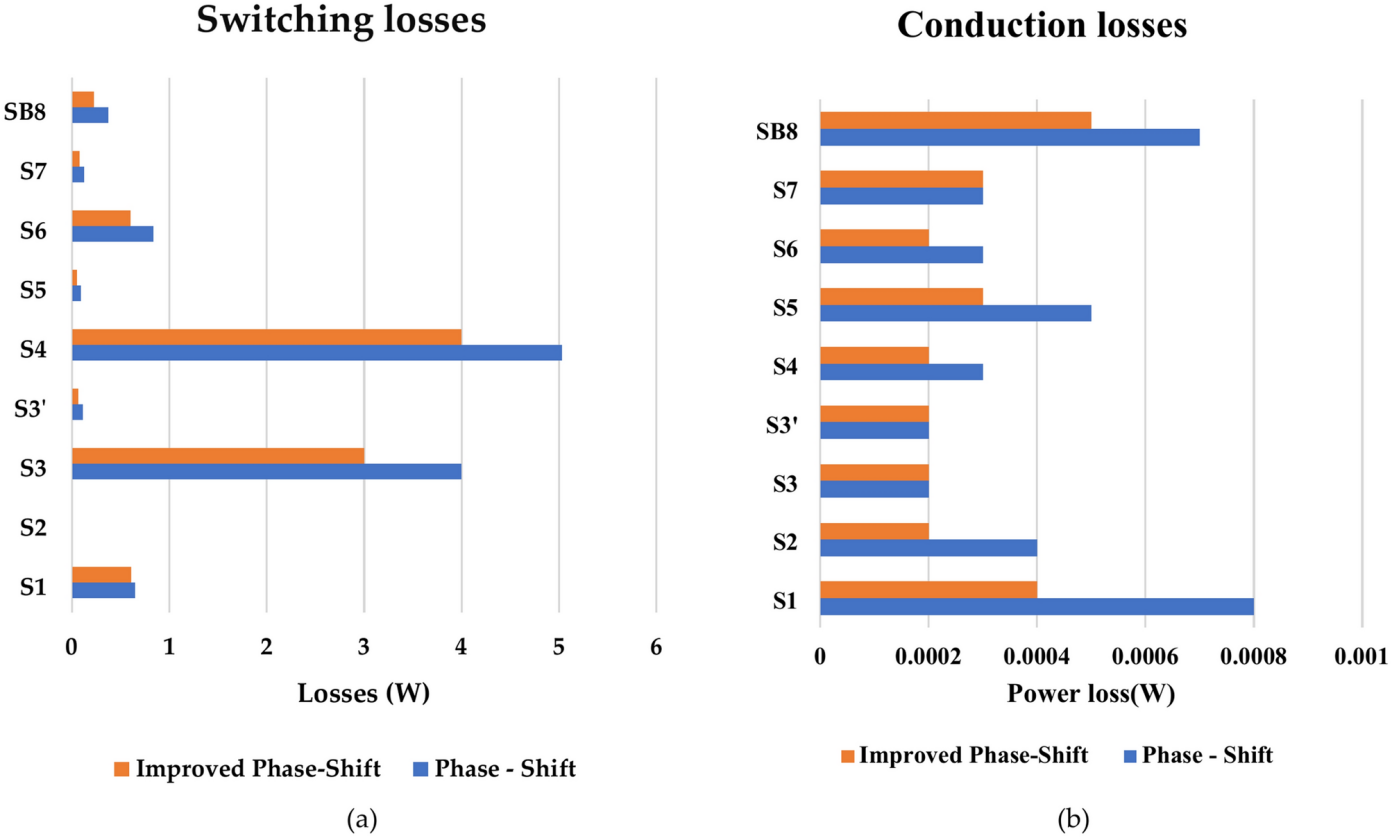
Switching and conduction losses implementing phase shift and improved phase shift.

Figure 9 shows the switching and conduction losses on 500W and switching frequency 10 kHz.

**Fig. 10:**
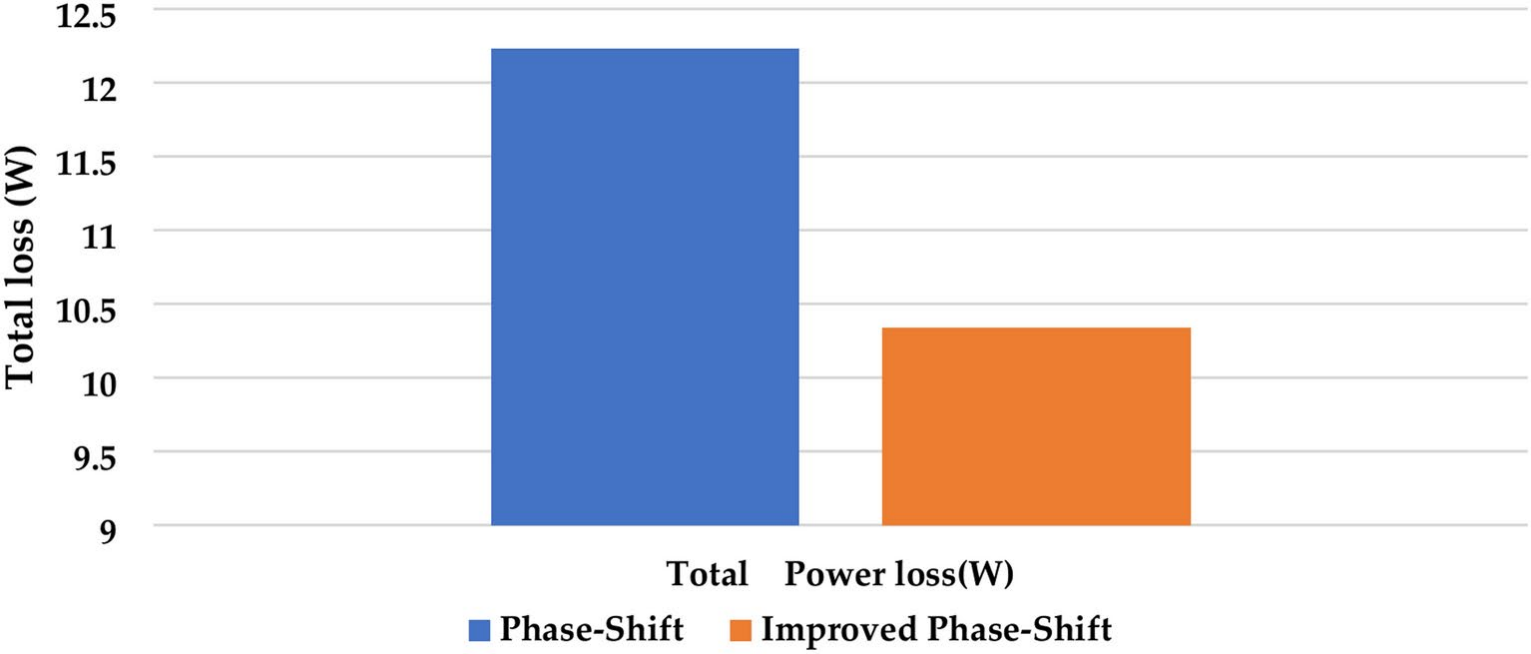
Total power loss.

Figure 10 compares the total power loss when implementing PS PWM and IPS PWM. Efficiency is one of the most important parameters for the analysis and application of an inverter. Efficiency vs Power curve is shown in Fig. 5. It can be seen in Fig. 5, that the maximum attainable efficiency is 98.05% at 150 W.

Figure 9 compares switching and conduction losses when implementing PS PWM and IPS PWM. It can be observed that switching losses have been reduced significantly while conduction losses either remain the same or are reduced.

The total power loss of switches is obtained as follows^10^:

10 $${\Delta }P_{hbt} = {\Delta }P_{hbt1} + {\Delta }P_{hbt2} + {\Delta }P_{hbt3} + {\Delta }P_{hbt4}$$

The power losses of the switches in the first, second, third and fourth switch legs are denoted as ΔP_hbt1_, ΔP_hbt2_, ΔP_hbt3_, and ΔP_hbt4_ respectively.

The expression for the power loss of a diode is:

11 $${\Delta }Pd = f_{sw} \int\limits_{Tch} {(vsd \cdot ich\left( t \right) + Rdn.ich\left( t \right)^{2} ) + f_{sw} \cdot E_{doff} }$$

The variables *Tch*, *vsd*, *ich(t)*, *Rdn*, *f*_*sw*_, and *Ed*_*off*_ show how long it takes for the diode to charge, the ON-state reverse voltage of the diode, instantaneous current, ON-state resistance of the diode, and the wasted energy due to the reverse recovery stage of the diode.

The power losses of the nth capacitor due to ESR is given as^10^:

12 $${\Delta }P_{capn} = f \cdot ESRn\smallint i\left( t \right) \cdot 2dt$$

The total power loss of the proposed inverter is given as follows:

13 $$\Delta Pt = \Delta P_{hbt} + 2\Delta {\text{Pd}}\mathop \sum \limits_{{{\text{n}} = 1}}^{4} \Delta {\text{P}}_{{{\text{capn}}}}$$

## Reliability analysis

**Fig. 11:**
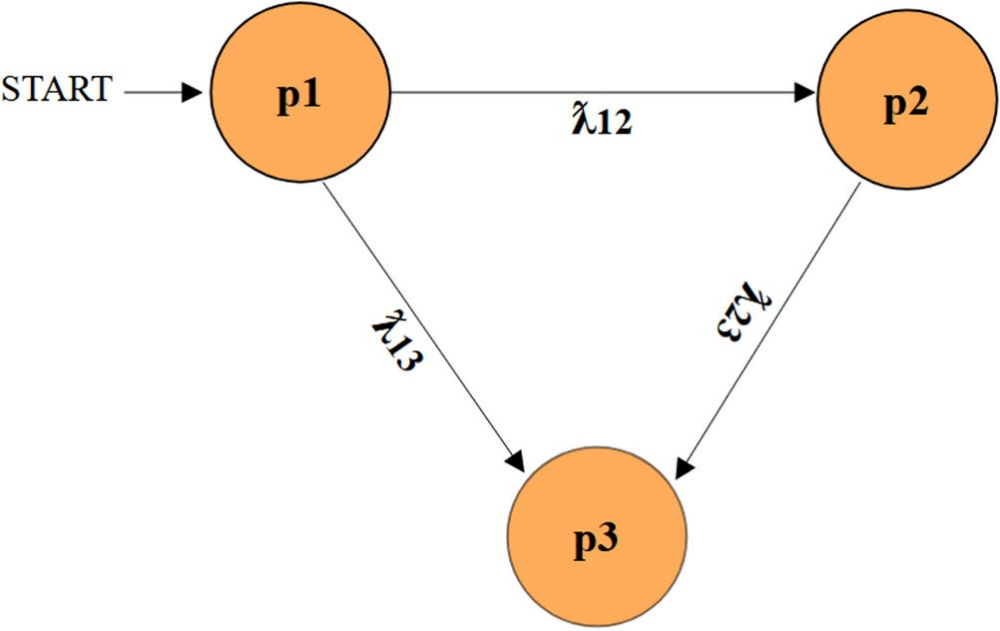
Markov chain model.

A measuring system’s stability, dependability, and consistency are evaluated using reliability analysis. Reliability analysis is a tool used by researchers and practitioners to assess the consistency and dependability of the instruments they use. A trustworthy measurement equipment can be recognized by its dependable and constant operation. The reliability of inverters is critical to the overall stability and efficacy of these systems. As long as the device is operated within its designated safe operating range, reliability is defined as the possibility that it will carry out its intended functions (such as turning on, off, and changing states) for a predetermined amount of time during normal operation. The Markov approach is a widely used method for assessing a system’s reliability. Because Markov chains are good at representing systems with probabilistic state transitions across time, they are frequently used in reliability analysis. The system under analysis is represented by Markov models in the Markov chain technique. Generally, this procedure comprises identifying discrete states (e.g., functioning properly, malfunctioning, or breaking down) in which the system is capable of existing and computing the likelihood of the system changing between these states. The suggested inverter’s Markov chain diagram is shown in Fig. 11.

The reliability analysis for the proposed inverter is performed at 500W load which gives efficiency of about 98.05%. The inverter is characterized by three states, namely P_1_, P_2_, and P_3_. The state p1 represents the condition in which all components are in good condition. The state p2 indicates a faulty condition where the inverter fails to produce any waveform levels due to the failure of one or two components. The state p3 represents a failure condition where the output fails to generate any waveform levels^11^. The mathematical definition of the following is possible using P1(t), P2(t), and ƛx, which represent the occupational probability of the state p1 and the failure rate of the element x, respectively**:**

14 $$\frac{d}{dt}\left[ {P_{1} \left( t \right)P_{2} \left( t \right)} \right] = \left[ {P_{1} \left( t \right)P_{2} \left( t \right)} \right]\left[ {\begin{array}{*{20}c} { - \lambda 10} & {\lambda 10} \\ 0 & 0 \\ \end{array} } \right]$$

The reliability of the proposed inverter can be written as:

15 $$R\left( t \right) = P_{1} \left( t \right) = {\text{exp}}\left[ { - \mathop \sum \limits_{i = 1}^{5} \lambda_{si} + \mathop \sum \limits_{i = 1}^{4} \lambda_{ci} + \lambda_{L} } \right]$$

The failure rate of each component can be defined as:

16 $$\lambda_{x} = \lambda_{b} \mathop \prod \limits_{i = 1}^{n} \alpha_{i}$$

The symbol λ_b_ represents the base failure rate of the component, which is 0.012 for the switch and 0.00254 for the capacitor^11^. The variable n represents the number of α factors that impact the failure rate of component x. These factors include temperature (α_T_), quality (α_Q_), application (α_A_), and environment (α_E_). As a result, the subsequent data delineates the rates at which a switch (λ_S_) and capacitor (λ_C_) fail):

17 $$\lambda_{S} = \lambda_{b} *\alpha_{T} *\alpha_{Q} *\alpha_{A} *\alpha_{E}$$

18 $$\lambda_{C} = \lambda_{b} *\alpha_{CV} *\alpha_{Q} *\alpha_{S} *\alpha_{E}$$

When assessing reliability, it is presumed that the semiconductor device is in perfect working order; thus, α_Q_ = α_A_ = 1. Moreover, α_E_ = 1 can be chosen under the assumption that all components function in an identical environment. All factors, with the exception of α_T_, are assumed to remain constant^11^. The computation of α_T_ for a semiconductor switch is as follows:

19 $$\alpha_{T}^{switch} = \exp \left[ { - 1925\left( {\frac{1}{Tj + 273} - \frac{1}{298}} \right)} \right]$$

where T_j_ is the device junction temperature and is determined using the following relations:

20 $$T_{j} = T_{C} + \theta_{jc} *P_{loss}$$

21 $$T_{C} = T_{a} + \theta_{ca} *P_{loss}$$

The variables denoted as *T*_*a*_,* θ*_*ca*_,* θ*_*jc*_ and Ploss are as follows: *θ*_*jc*_ represents the thermal impedance of the switch, which is assumed to be 0.45 °C/W; *θ*_*ca*_ represents the case-to-ambient thermal impedance, which is 62 °C/W; T_a_ is assumed to 25 °C^11^and P_loss_ signifies the overall power loss incurred by the switch. The computation of the capacitance factor α_CV_ and stress factor α_S_ for the capacitor is as follows:

22 $$\alpha_{CV} = 0.34*C^{0.012}$$

23 $$\alpha_{S} = V_{S}^{2.43}$$

In this case, the operating voltage to rated voltage ratio is represented by V_s_, while the capacitance, expressed in microfarads, is indicated by C. Utilizing PLECS, a thorough thermal analysis of the inverter topology of study is carried out. Table 5 shows power losses obtained in PLECS, ${\varvec{\alpha}}_{{\varvec{T}}}^{{{\varvec{switch}}}}$ and ${\varvec{\lambda}}_{{\user2{si }}}$ of the switches in a healthy state.

**Table 5:** Failure rates of each switch at healthy state.

Switches	Healthy state		
	Power loss (W)	${\varvec{\alpha}}_{{\varvec{T}}}^{{{\varvec{switch}}}}$	${\varvec{\lambda}}_{{\user2{si }}}$(10^−2^)
S_1_	0.3044	1.35	1.60
S_2_	0.0005	1.00	1.00
S_3_	3.0584	15.08	18.01
S_3’_	0.0756	1.08	1.29
S_4_	4.2134	20.32	24.41
S_5_	0.0657	1.08	1.29
S_6_	0.6709	2.05	2.20
S_7_	0.0823	1.12	1.40
S_B8_	0.2500	1.32	1.58

Regarding the Markov chain in Figure 8, the corresponding probabilities are calculated as:

24 $$\frac{d}{dt}\left[ {P_{1} \left( t \right)P_{2} \left( t \right)P_{3} \left( t \right)} \right] = \left[ {P_{1} \left( t \right)P_{2} \left( t \right)P_{3} \left( t \right)} \right]*\left[ A \right]$$

25 $$\begin{aligned} & \lambda_{12} = \lambda_{S1} + \lambda_{S2} + \lambda_{S5} + \lambda_{S7} + \lambda_{SB8} + \lambda_{S6} + \lambda_{S1} *\lambda_{S2} + \lambda_{S1} *\lambda_{S5} + \lambda_{S1} *\lambda_{S6} + \lambda_{S1} *\lambda_{S7} + \lambda_{S1} *\lambda_{SB8} \\ & \quad + \lambda_{S2} *\lambda_{S5} + \lambda_{S2} *\lambda_{S6} + \lambda_{S2} *\lambda_{S7} + \lambda_{S2} *\lambda_{SB8} + \lambda_{S5} *\lambda_{S6} + \lambda_{S5} *\lambda_{S7} + \lambda_{S5} *\lambda_{SB8} + \lambda_{S7} *\lambda_{S6} + \lambda_{SB8} *\lambda_{S6} \\ & \quad + \lambda_{S7} *\lambda_{SB8} + \lambda_{C1} *\lambda_{C3} + \lambda_{C1} + \lambda_{C3} \\ \end{aligned}$$

26 $$\lambda_{13} = \lambda_{S3} + \lambda_{S3^{\prime}} + \lambda_{S4} + \lambda_{S3} *\lambda_{S4} + \lambda_{S3} *\lambda_{{S3^{\prime}}} + \lambda_{{S3^{\prime}}} *\lambda_{S4} + \lambda_{C2} + \lambda_{C4} + \lambda_{C4} *\lambda_{C2}$$

27 $$\begin{aligned} \lambda_{23} & = \lambda_{S1} *\lambda_{S3} + \lambda_{S1} *\lambda_{S4} + \lambda_{S1} *\lambda_{{S3^{\prime}}} + \lambda_{S2} *\lambda_{S3} + \lambda_{S2} *\lambda_{S4} + \lambda_{S2} *\lambda_{{S3^{\prime}}} + \lambda_{S3} *\lambda_{S5} + \lambda_{S3} *\lambda_{S6} \\ & \quad + \lambda_{S3} *\lambda_{S7} + \lambda_{S3} *\lambda_{SB8} + \lambda_{{S3^{\prime}}} *\lambda_{S6} + \lambda_{{S3^{\prime}}} *\lambda_{S5} + \lambda_{{S3^{\prime}}} *\lambda_{S7} + \lambda_{{S3^{\prime}}} *\lambda_{SB8} + \lambda_{S4} *\lambda_{S5} \\ & \quad + \lambda_{S4} *\lambda_{S6} + \lambda_{S4} *\lambda_{S7} + \lambda_{S4} *\lambda_{SB8} + \lambda_{C1} *\lambda_{C4} + \lambda_{C1} *\lambda_{C2} \\ \end{aligned}$$

28 $$\left[ A \right] = \left[ {\begin{array}{*{20}c} { - (\lambda_{12} + \lambda_{13} )} & {\lambda_{12} } & {\lambda_{13} } \\ 0 & { - \lambda_{23} } & {\lambda_{23} } \\ 0 & 0 & 0 \\ \end{array} } \right]$$

Table 5 shows the power loss and failure rated of individual switches at healthy state. The matrix [A] calculates the values based on the state transfers of switches, specifically from a healthy state to a fault state, from a healthy state to a failure state, and from a fault state to a failure state. All of these statuses are taken into account for every switch and all potential pairings of two switches, in the event of a simultaneous failure.

The final $\left[ {\varvec{A}} \right]$ is as follows:

29 $$\left[ A \right] = \left[ {\begin{array}{*{20}c} { - 6.96*10^{ - 2} } & {3.13*10^{ - 2} } & {3.83*10^{ - 2} } \\ 0 & { - 125.4*10^{ - 2} } & {125.4*10^{ - 2} } \\ 0 & 0 & 0 \\ \end{array} } \right]$$

The resulting equations are as follows:

30 $$P_{1} \left( t \right) = {\text{exp}}\left( { - 6.96*10^{ - 2} } \right)t$$

31 $$P_{2} \left( t \right) = 1.67\left( {\exp \left( { - 125.4*10^{ - 2} } \right)t - \exp \left( { - 6.96*10^{ - 2} } \right)t} \right)$$

32 $$P_{3} \left( t \right) = 1.16\exp \left( { - 6.96*10^{ - 2} } \right)t - 10.74\exp \left( { - 125.4*10^{ - 2} } \right)t$$

33 $$R\left( t \right) = P_{1} \left( t \right) + P_{2} \left( t \right) + P_{3} \left( t \right)$$

34 $$= 0.49\exp \left( { - 6.96*10^{ - 2} } \right)t - 9.07{\text{exp}}\left( { - 125.4*10^{ - 2} } \right)t$$

Mean Time to Failure (MTTF) or Mean Time before Failure (MTBF):

35 $${\text{MTTF}}/{\text{MTBF}} = \mathop \smallint \limits_{0}^{\infty } {\text{R}}\left( {\text{t}} \right) = 0.175{*}10^{6} \left( {\text{h}} \right) = 20.04{\text{ years}}$$

### Reliability comparison with literature

This section compares the reliability of different topologies in terms of Mean Time to Failure (MTTF) or Mean Time Before Failure (MTBF).

For^34^

36 $$R\left( t \right) = 1.9\exp \left( { - 10.43} \right)t - 0.83\exp \left( { - 16.27} \right)t - 0.08\exp \left( { - 18} \right)t$$

37 $${\text{MTTF}}/{\text{MTBF}} = \mathop \smallint \limits_{ 0}^{\infty } R\left( t \right) = 0.126*10^{6} \left( h \right) = 14.3\;years$$

For^35^

38 $$R\left( t \right) = 5.09\exp \left( { - 10.43} \right)t + 1.49\exp \left( { - 18} \right)t - 5.55\exp \left( { - 13.43} \right)t$$

39 $${\text{MTTF}}/{\text{MTBF}} = \mathop \smallint \limits_{0}^{\infty } R\left( t \right) = 0.157*10^{6} \left( h \right) = 17.9\;years$$

For^36^

40 $$R\left( t \right) = 4.23\exp \left( { - 18.83} \right)t - 3.23exp\left( { - 22.92} \right)t$$

41 $${\text{MTTF}}/{\text{MTBF}} = \mathop \smallint \limits_{0}^{\infty } R\left( t \right) = 0.08*10^{6} \left( h \right) = 10.2\;years$$

From Eqs. (37), (39) and (41), showing the MTBF in years and hours, when compared to (35), it can be observed that the reliability of the proposed inverter is better. The topology proposed in^34^ used 16 switches, the one^35^ utilizes 12 and the one in^36^ utilizes 16. It can be noted that^35^ utilizing lesser number of switches has better reliability. The proposed topology utilizes 10 power switches in total and thus enhancing reliability. The reliability function, Eq. (34), for the proposed topology is for a seven level, furthermore, no redundant switch is required in the proposed topology, which implies no switch remains unutilized during healthy operation implying full utilization of all switches, thus resulting in higher reliability. The proposed topology’s reliable function solution is more superior.

## Simulation and experimental results

The performance parameters of the proposed inverter are assessed by conducting a laboratory experiment and using a MATLAB/Simulink model. The simulation model incorporates the integration of the proposed inverter with the PV unit using a PI controller. The effectiveness of the suggested inverter and modulation technique is evaluated using a load that can be easily accessed within the experiment.

### Simulation results

**Fig. 12:**
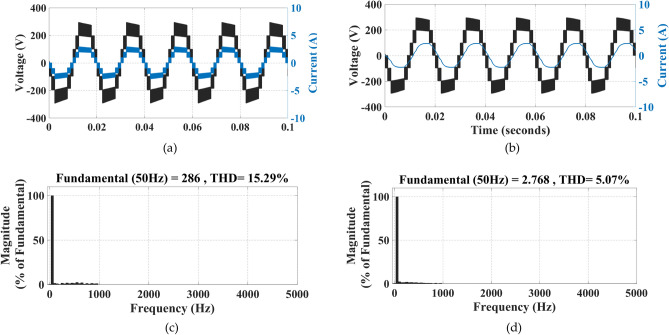
Implementing IPS PWM: (**a**) current and voltage waveforms at R load, (**b**) current and voltage waveforms at RL load, (**c**) THD (%) of voltage, (**d**) THD (%) of current.

**Fig. 13:**
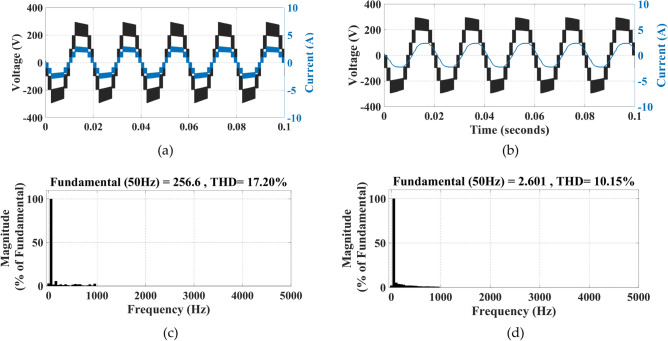
Implementing PS PWM: (**a**) current and voltage waveforms at R load, (**b**) current and voltage waveforms at RL load, (**c**) THD (%) of voltage, (**d**) THD (%) of current.

The simulation of the two modulation techniques is performed, their waveforms and THD(%) are shown in Figs. 12 and 13.

It can be clearly observed that the THD (%) when implementing IPS PWM has been significantly reduced.

Further simulation results are taken while implementing IPS PWM.

Table 6 shows the specifications of the parameters used for obtaining simulation results.

**Table 6:** Specification for simulation.

Parameters	Specifications
Switching frequency	10 kHz
Voltage frequency	50 Hz
R load	100 Ω
RL load	100Ω, 200 mH
C_1 ,_ and C_2_	1000 µF
C_3_ and C_4_	2200 µF
Input voltage	100 V
Modulation index	1

**Fig. 14:**
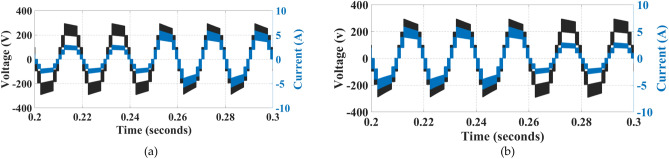
Waveforms under varying R load: (**a**) R load increasing, (**b**) R load decreasing.

Figure 14 shows the waveforms when varying R load simultaneously. This shows the efficient working of the proposed topology, working under simultaneously varying R load.

**Fig. 15:**
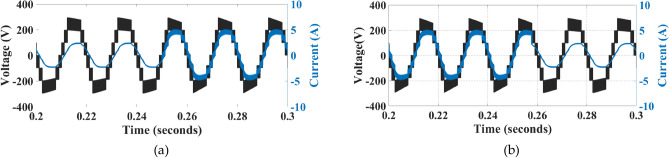
Waveforms under varying RL load: (**a**) RL load increasing, (**b**) RL load decreasing.

Figure 15 shows the waveforms when varying RL load simultaneously. This shows the efficient working of the proposed topology under simultaneously varying RL load.

**Fig. 16:**
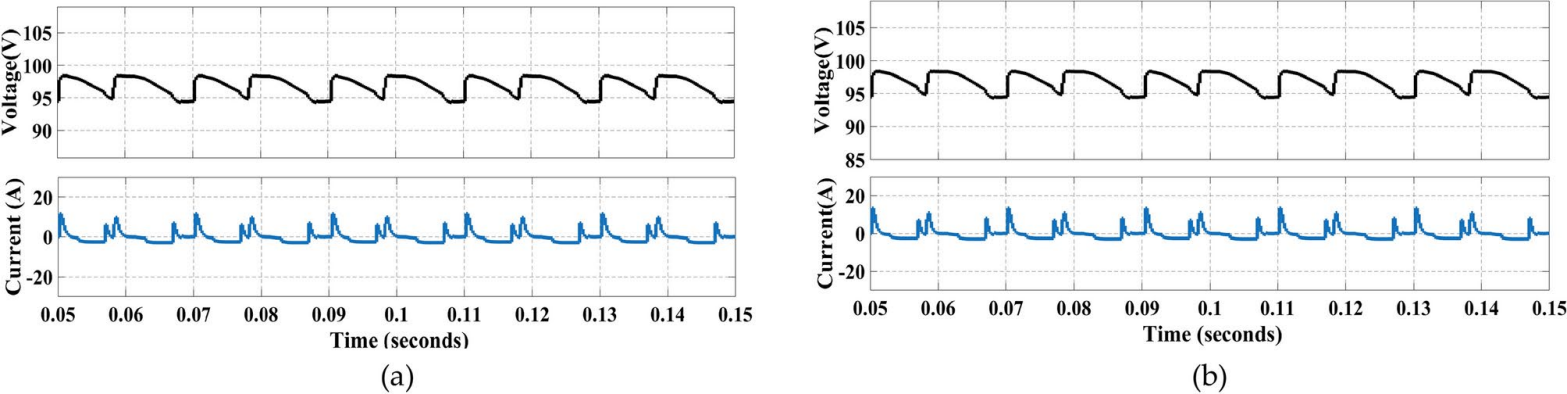
Voltage and current waveforms: (**a**) Through C_1_, (**b**) Through C_2_.

Figure 16 shows the voltage and current waveforms of capacitors C_1_ and C_2_.

**Fig. 17:**
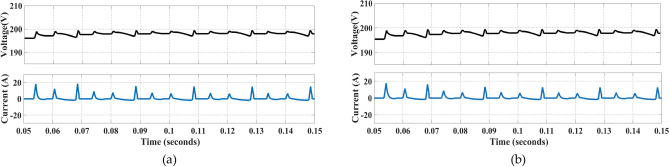
Voltage and current waveforms: (**a**) Through C_3_ and (**b**) Through C_4_.

Figure 17 shows the voltage and current waveforms through capacitors C_3_ and C_4_.

**Fig. 18:**
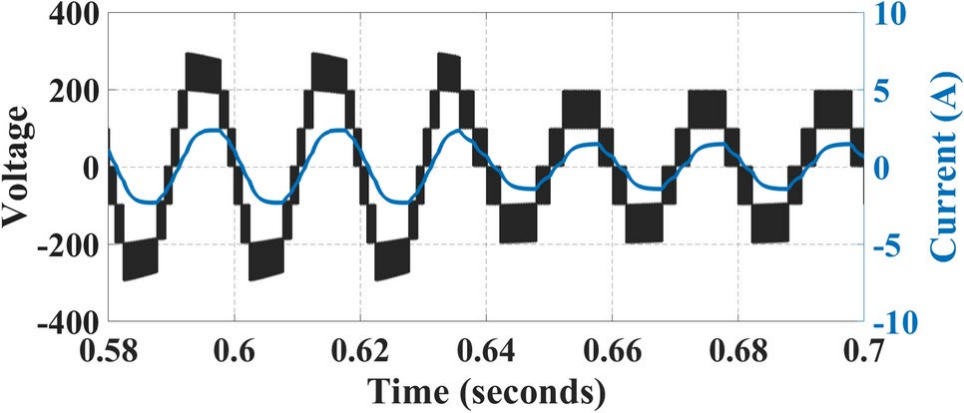
Modulation index being varied.

Figure 18 shows the current and voltage waveforms when the modulation index is simultaneously varied.

### Experimental results

**Fig. 19:**
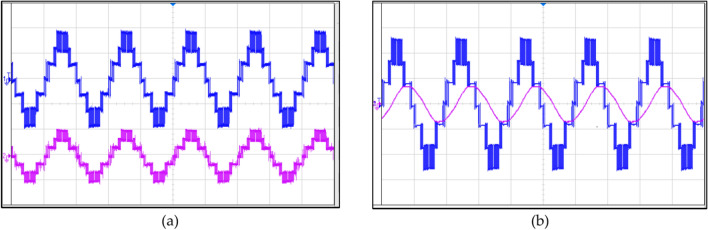
Voltage and current waveforms: (**a**) at R-load, (**b**) at RL-load.

A This section uses an experimental setup to assess the proposed topology. The outcomes of the suggested topology are validated through an experimental configuration conducted within the laboratory and experimentally observed voltage and current waveforms under R load and RL load, where R = 40 Ω and L = 120 mH are depicted in Fig. 19a and b.

A seven-level stepped waveform is generated at power factor unity for voltage at both R load and RL load, whereas a sinusoidal wave is generated for RL load and a stepped seven level is generated for current at R load.

Table 7 shows the specifications of the parameters used for obtaining the results.

**Table 7:** Specification for experiment.

Parameters	Specifications
R load	40 Ω
RL load	40 Ω, 120 mH
Switching frequency	20 kHz
Modulation index	1
Input voltage (V_o_)	50

**Fig. 20:**
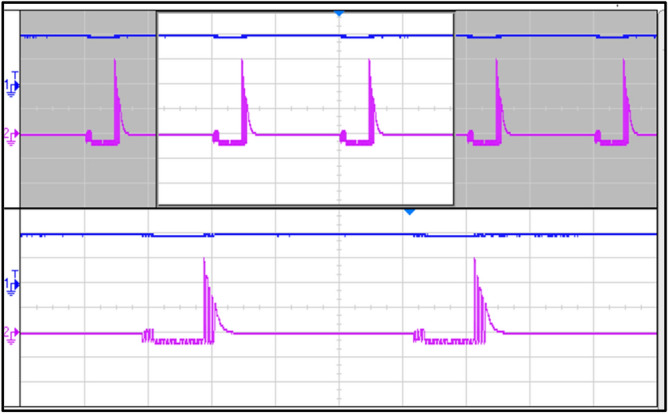
Voltage and current waveforms through a capacitor.

Figure 20 shows the voltage and current waveforms through capacitors.

**Fig. 21:**
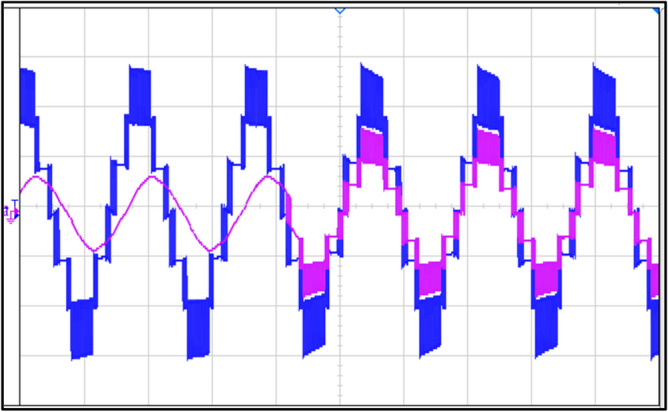
Load switched from RL–R Load.

Figure 21 shows the waveforms when the load is switched from RL load to R load. This shows that the inverter worked for both R and RL loads even when the loads were being switched simultaneously.

**Fig. 22:**
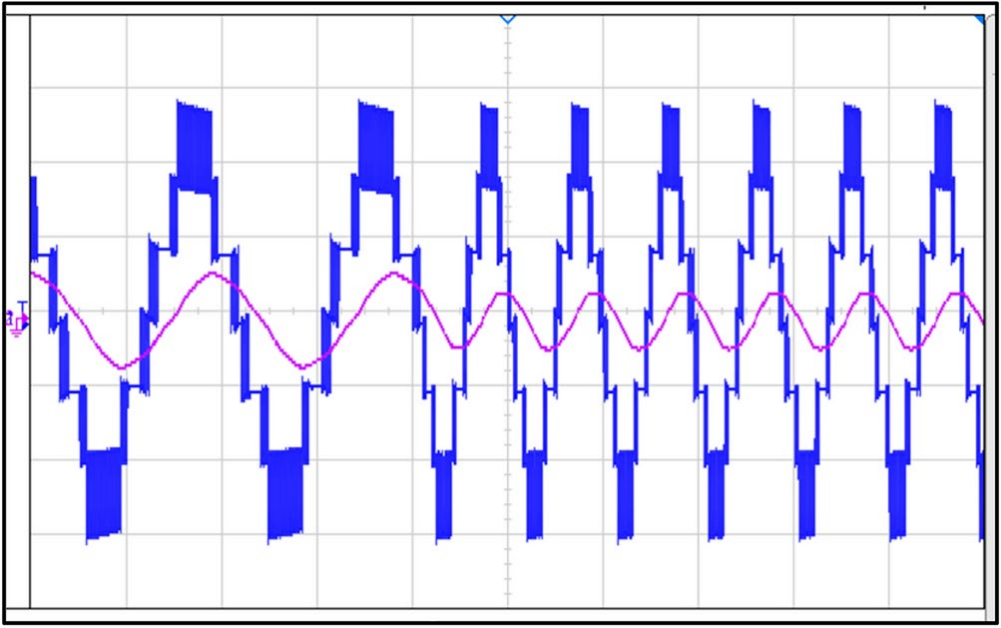
Frequency switched from 50 to 100 Hz.

Figure 22 shows the waveforms when the frequency is switched from 50 to 100 Hz at power factor unity. This shows the capability of the inverter to work accurately at different frequencies.

**Fig. 23:**
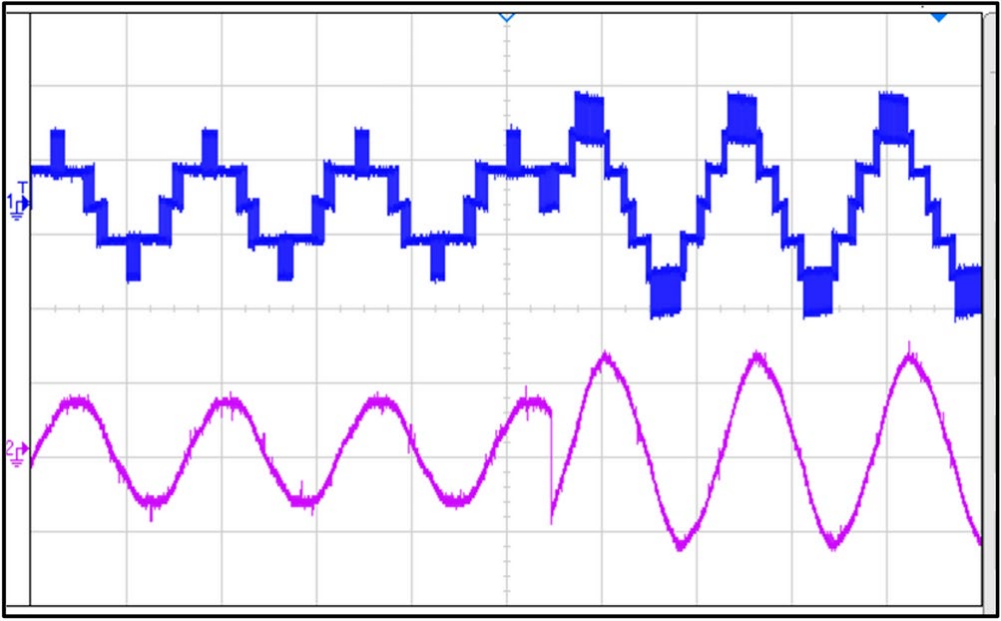
Modulation index increasing from 0.7 to 1.

Figure 23 shows the voltage and current waveforms when the modulation index increases from 0.7 to 1. This shows a reduction in levels in voltage stepped waveform, from seven to five levels and a change in amplitude of the current sinusoidal waveform.

**Fig. 24:**
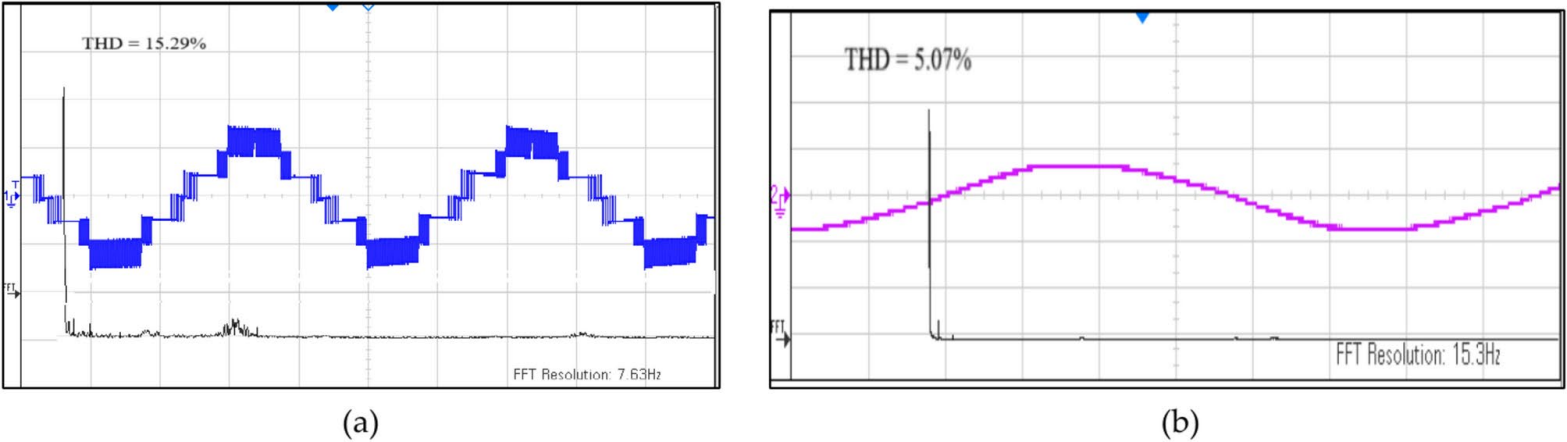
(**a**) Experimental results for THD% in Voltage waveform. (**b**) Experimental results for THD% in Current waveform.

Figure 24 shows the experimental results of voltage and current harmonics.

All the results are taken for load R = 40 Ω and L = 120 mH. The above results show the accuracy and efficiency of the proposed topology and the modulation scheme. All the results are taken for the proposed modulation scheme implemented on the proposed topology, hence validating its working and efficiency.

## Conclusion

This article proposes a single-phase CGT inverter generating seven levels, utilizing fewer switches and thus reducing component count, making it less bulky, when compared to seven level MLIs. This article also proposes an Improved Phase Shift (IPS) PWM which reduces THD (%) by 5% for current and by 2% for voltage when compared to original PS PWM, reduces losses and thus enhances efficiency. It is evident through simulation and experimental results that when IPS, when implemented on the proposed topology, there are lower losses and thus the efficiency increases. The proposed inverter was shown to be possible and workable through simulations, tests, and an analysis of power loss in PLECS.

## Data Availability

The datasets used and/or analysed during the current study available from the corresponding author on reasonable request.
